# It Steps down from Its Airy Throne

**Published:** 1879-09-20

**Authors:** H. W. Carpenter

**Affiliations:** California


### *IT STEPS DOWN FROM ITS AIRY THRONE.*

BY H. W. CARPENTER, M.D., OF CALIFORNIA.

What wonders that youngest of the sciences, chemistry, is disclosing to the scientific world! All the so-called permanent gases, compound and elementary, have now been conquered, disestablished, brought down from their airy throne to the liquid form, and from the liquid it is but a step to the solid, thus proving the three forms of matter to be but widely separated states of the same condition—accidents of temperature. Cold and pressure, under the guidance of those master minds, M. Ravel Pictet and M. Cailletet, have liquefied them all, from hydrogen down to marsh gas; while the lightest of them all—hydrogen—has already been solidified, the little globules rattling down in the pan like shot on the floor, thus establishing the further fact that hydrogen is a mineral.

All matter may now be demonstrated to exist in three forms—as light, heat and electricity are synonymous terms—different degrees of the same agent. Our manuals of chemistry teaching that matter exists in upwards of sixty forms, may now be laid in the tomb of oblivion.

After all, is there more than *one* form of matter? I think not. Oxigen gave the most trouble in the process of liquefaction. It resisted a pressure of eight hundred atmospheres; but when conducted in a small tube, surrounded by a larger one, through which a double current of sulphurous acid and carbonic acid was circulating, it liquefied under a pressure of three hundred and twenty atmospheres.

Is the importance of the liquefied gases appreciated in a therapeutic point of view? Do the profession generally understand that these gases possess the power of at once destroying all cryptogamic, infusorial, vegetative, fermentative life? Oxygen under pressure destroys at once all of these; but such poisons as owe their power to chemical compounds resist it.

Nothing is better established in pathology than that zymotic diseases are spore, or bacteria in etiology. For the cure of this class of diseases no other remedy is worthy to be canvassed by the side of sulphurous acid. A diphtheritic membrane will dissolve and disappear in a ten per cent solution of the acid wtth the rapidity of a lump of sugar in a tumbler of water; while administered internally in diphtheria it destroys the bacteria in the blood with the same degree of certainty that an alkali gradually added to an acid solution brings it to a neutral state.

As the liquefied acid sulphurous is compatible with the tinc. ferri mur., potassa chlo., etc., it can be added to the usual chlorine treatment by the timid, if desired, but it is useless. I prescribe as follows:

℞ Acid sulphurosii........................................ ʒ vj.

Glycerine........................................ ℥ iij.

Sol. potassa chlo. sat. ad.................................. ℥ viij. M

Dose, a teaspoonful to two or three, according to age, often repeated. Nothing else.

